# Uncovering the Pharmacological Mechanism of Stemazole in the Treatment of Neurodegenerative Diseases Based on a Network Pharmacology Approach

**DOI:** 10.3390/ijms21020427

**Published:** 2020-01-09

**Authors:** Jing Zhang, Huajun Li, Yubo Zhang, Chaoran Zhao, Yizi Zhu, Mei Han

**Affiliations:** Key Laboratory of Radiopharmaceuticals, Ministry of Education, College of Chemistry, Beijing Normal University, Beijing 100875, China; zhangjing0532@126.com (J.Z.); lihuajun726@foxmail.com (H.L.); zhangyb1995@163.com (Y.Z.); 201821150104@mail.bnu.edu.cn (C.Z.); zhuyizi1112@163.com (Y.Z.)

**Keywords:** stemazole, neurodegenerative diseases, anti-apoptosis, network pharmacology, molecular mechanisms

## Abstract

Stemazole exerts potent pharmacological effects against neurodegenerative diseases and protective effects in stem cells. However, on the basis of the current understanding, the molecular mechanisms underlying the effects of stemazole in the treatment of Alzheimer’s disease and Parkinson’s disease have not been fully elucidated. In this study, a network pharmacology-based strategy integrating target prediction, network construction, gene ontology (GO) and Kyoto Encyclopedia of Genes and Genomes (KEGG) pathway enrichment analyses, and molecular docking was adopted to predict the targets of stemazole relevant to the treatment of neurodegenerative diseases and to further explore the involved pharmacological mechanisms. The majority of the predicted targets were highly involved in the mitogen-activated protein kinase (MAPK) signaling pathway. RAC-alpha serine/threonine-protein kinase (AKT1), caspase-3 (CASP3), caspase-8 (CASP8), mitogen-activated protein kinase 8 (MAPK8), and mitogen-activated protein kinase 14 (MAPK14) are the core targets regulated by stemazole and play a central role in its anti-apoptosis effects. This work provides a scientific basis for further elucidating the mechanism underlying the effects of stemazole in the treatment of neurodegenerative diseases.

## 1. Introduction

Neurodegenerative diseases, which comprise a type of chronic and progressive disease characterized by progressive degeneration of the structure and function of the nervous system [1], have become the most feared maladies in older people and one of the most serious healthcare problems worldwide [2]. Alzheimer’s disease (AD) and Parkinson’s disease (PD) are two common neurodegenerative diseases that may lead to cognitive decline, memory loss, and movement disorders [3,4]. A total of 50 million people worldwide were living with AD as of 2018. This number will rise to approximately 152 million by 2050 [5]. According to the Global Burden of Disease Study, there are currently 6.2 million individuals suffering from PD, and the incidence of PD has surpassed that of AD [6].

Unfortunately, despite tremendous effort and expenditures, the existing clinical treatments for neurodegenerative diseases result in only temporary and limited symptomatic relief [7,8], and they are unable to treat the root causes or delay disease progression [9,10]. Therefore, there is an urgent need for effective therapeutic strategies based on the understanding of the pathological mechanisms underlying neurodegenerative diseases. Progressive neuronal damage or loss is one of the deterministic features of neurodegenerative diseases such as AD and PD [11,12]. Stem cell-based therapy has exciting potential for neuroprotection [13,14]. The loss of brain function caused by injury and aging can be treated by mobilizing the proliferation and differentiation of endogenous stem cells and replacing them with exogenous stem cells [15,16].

Recently, a novel small molecule with the ability to regulate stem cells has shown potential for the treatment of neurodegenerative diseases. Stemazole (ST) was discovered by a high-throughput screen [17]. We previously demonstrated that stemazole can prevent several types of stem cells from undergoing apoptosis under nutritional deprivation and injury conditions, including human hippocampal stem cells, pancreatic stem cells, cardiac stem cells [17], and human embryonic stem cells [18]. Our experimental evidence also revealed the neuroprotective effects of stemazole in animal models of neurodegenerative diseases. Stemazole exhibited a therapeutic effect in a beta-amyloid (Aβ) injection rat model of Alzheimer’s disease [19] and a protective effect on the impaired dopamine system in a 1-methyl-4-phenyl-1,2,3,6-tetrahydropyridine (MPTP)-induced acute mouse model of Parkinson’s disease [20]. In addition, stemazole has high absolute oral bioavailability, which makes it possible to develop it into an oral preparation. It can cross the blood–brain barrier and shows stable accumulation in the brain [21].

These studies have indicated that stemazole is a promising drug candidate. However, the regulatory mechanism underlying the effects of stemazole against AD and PD has not been systematically elucidated. Studies of the molecular targets and relevant signal pathways will provide a better understanding of the effects of stemazole in the treatment of neurodegenerative diseases.

Network pharmacology is an innovative method to study the mechanisms of drugs at the systemic level [22]. It encompasses bioinformatics, network analysis, and experimental approaches and integrates multiple sources of information [23]. Therefore, network approaches can accurately discriminate potential drug–target interactions [24].

Using the network pharmacology strategy, our study systematically investigated the potential targets and molecular mechanisms underlying the effects of stemazole against neurodegenerative diseases. First, we predicted the molecular targets of stemazole through chemical similarity analysis, pharmacophore model screening, and reverse docking. Pathological targets were identified using various bioinformatics platforms. We then performed enrichment analysis according to gene ontology (GO) and Kyoto Encyclopedia of Genes and Genomes (KEGG) terms and constructed a drug–target–pathway network. Finally, molecular docking was used to verify the interactions between stemazole and its targets.

## 2. Results

### 2.1. Screening of Potential Targets

A database of targets associated with neurodegenerative diseases and the targets of stemazole was constructed. A total of 1981 and 1063 targets associated with AD and PD, respectively, were identified using the DisGeNET database v6.0 (Table S1). A total of 559 predicted targets of stemazole were obtained from PharmMapper, Drug Repositioning and Adverse Reaction via Chemical-Protein Interactome (DRAR-CPI), Drug Positioning and Drug Repositioning via Chemical-Protein Interactome (DPDR-CPI), TargetNet, and ChemMapper after eliminating duplicates (Table S2). The intersection of the three lists identified 91 therapeutic targets of stemazole related to both AD and PD. To further identify the therapeutic targets involved in the regulation of stem cells, 91 targets in the DrugBank database, UniProt, the Human Protein Atlas, the Comparative Toxicogenomics Database, and KEGG were analyzed. Twenty-nine targets were found to be involved in the processes of apoptosis and were putative therapeutic targets of stemazole, mediating its effects against neurodegenerative diseases through an anti-apoptosis mechanism, as shown in Table 1.

### 2.2. Protein–Protein Interaction (PPI) Network Construction and Analysis

**Figure 1:**
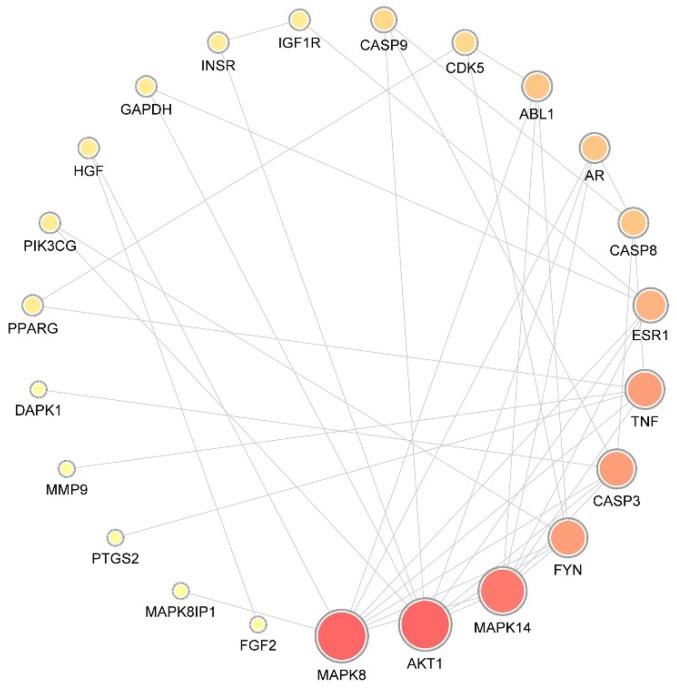
Protein–protein interaction network. The size of the circle represents the node degree of the target protein. MAPK, mitogen-activated protein kinase; AKT1, RAC-alpha serine/threonine-protein kinase; FYN, tyrosine-protein kinase Fyn; CASP, caspase; ESR, estrogen receptor; AR, androgen receptor; TNF, tumor necrosis factor; ABL1, tyrosine-protein kinase ABL1; CDK, Cyclin-dependent-like kinase; IGF1R, insulin-like growth factor 1 receptor; INSR, insulin receptor; GAPDH, glyceraldehyde-3-phosphate dehydrogenase; HGF, hepatocyte growth factor; PIK3CG, hosphatidylinositol 4,5-bisphosphate 3-kinase catalytic subunit gamma isoform; PPARG, peroxisome proliferator-activated receptor gamma; DAPK, death-associated protein kinase; MMP, matrix metalloproteinase; PTG, prostaglandin G/H synthase; FGF, fibroblast growth factor.

A total of 29 putative targets were uploaded to the STRING database to identify the functional partnerships and interactions between them. Protein interactions with a confidence score of 0.9 or higher were then imported into Cytoscape v3.7.1 to generate the protein–protein interaction (PPI) network, which comprised 23 nodes and 42 edges (Figure 1).

To identify the hub nodes and essential proteins in the PPI network, the topological parameters of the node degree were calculated by Network Analyzer, and the three centralities (betweenness, closeness, and subgraph) were determined by the CytoNCA plugin, as shown in Table 2. On the basis of the overlap between the top 10 proteins in each of the four groups, mitogen-activated protein kinase 8 (MAPK8), RAC-alpha serine/threonine-protein kinase (AKT1), MAPK14, tyrosine-protein kinase Fyn (FYN), caspase-3 (CASP3), estrogen receptor (ESR1), androgen receptor (AR), and CASP8 were identified as essential proteins with significant centrality values based on the network topology analysis.

### 2.3. GO and KEGG Pathway Enrichment Analyses

**Figure 2:**
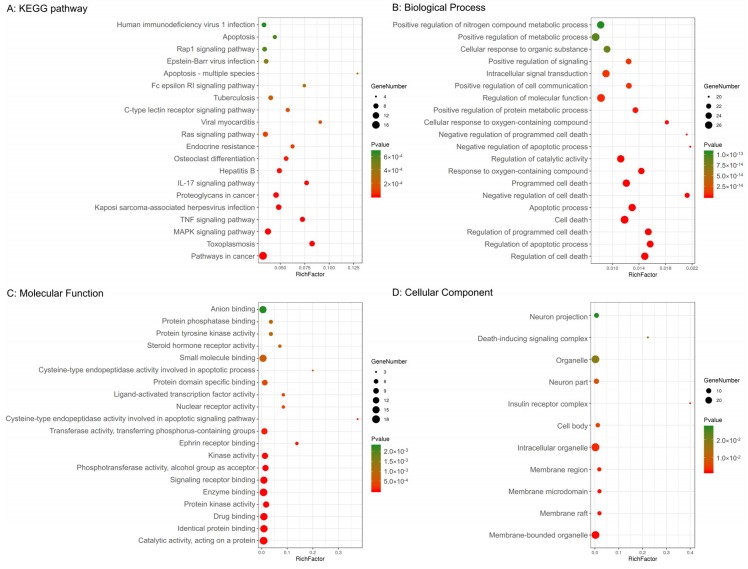
Kyoto Encyclopedia of Genes and Genomes (KEGG) pathway and gene ontology enrichment analyses of 29 target proteins (*p*-value ≤ 0.05). (**A**) The top 20 KEGG pathways. (**B**) The top 20 biological processes. (**C**) The top 20 molecular functions. (**D**) Eleven cellular components. The color scales indicate the different thresholds for the *p*-values, and the sizes of the dots represent the number of genes corresponding to each term.

In addition, 29 potential targets were submitted to the g: Profiler server for KEGG pathway annotation and GO enrichment analysis. The KEGG pathways and gene ontology terms with a *p*-value ≤0.05 were significantly enriched. The top 20 components were graphed using the OmicShare cloud platform (Figure 2).

**Figure 3:**
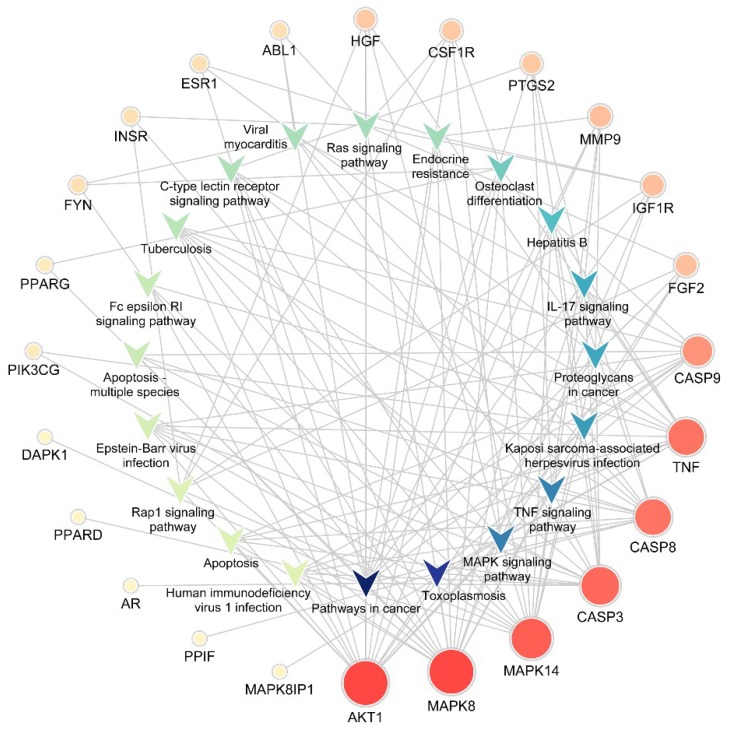
Target-pathway network. The V-shaped patterns represent pathways, and the circles represent targets. The sizes of the circles represent the degree of importance in the pathway. IL, Interleukin.

**Figure 4:**
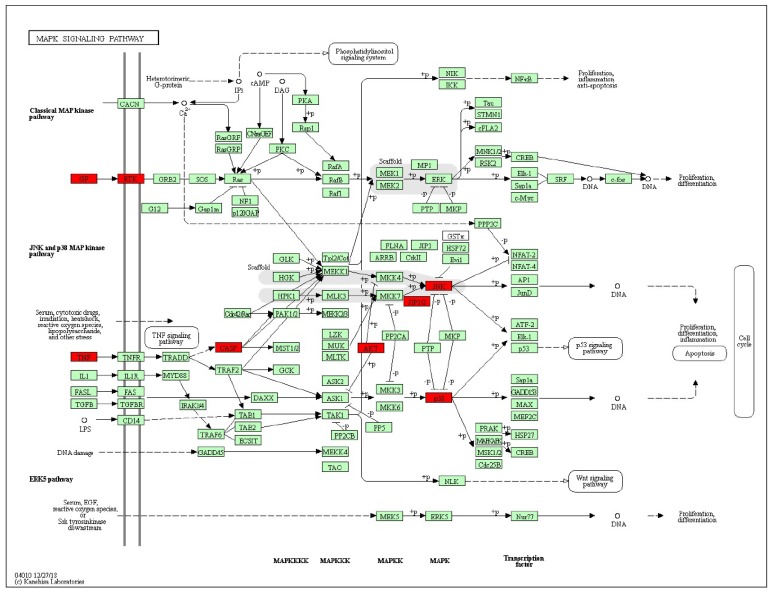
Representation of the targets of stemazole (ST) involved in the MAPK signaling pathway. The pathway maps were constructed using the KEGG mapper. The green rectangles and red rectangles represent unidentified proteins and identified proteins, respectively.

The KEGG pathway annotation showed that 27 of the 29 (93.1%) potential targets were significantly enriched in 53 pathways (Table S3). The statistical analysis indicated that 7 proteins were involved in the top 20 pathways (Figure 3) very frequently (≥10 times), which indicates that they are of great importance in the enriched pathways. The seven core proteins were MAPK8, AKT1, MAPK14, CASP3, CASP8, TNF, and CASP9. Among the enriched pathways, the MAPK signaling pathway was found to be dysregulated in neurodegenerative diseases. The predicted targets involved in the MAPK signaling pathway are shown in red in Figure 4.

The GO terms comprised three categories: biological process, molecular function, and cellular component. As shown in Figure 2, the top five biological processes included the regulation of cell death, regulation of apoptotic processes, regulation of programmed cell death, cell death, and apoptotic processes. The top five molecular functions included catalytic activity acting on a protein, identical protein binding, drug binding, protein kinase activity, and enzyme binding. The top five cellular components included membrane-bound organelles, membrane rafts, membrane microdomains, membrane regions, and intracellular organelles.

### 2.4. Molecular Docking

Docking studies were carried out between stemazole and five select important targets, AKT1, CASP3, CASP8, MAPK8, and MAPK14. These targets were chosen because not only were they key nodes of the PPI network, but they also played an important role in KEGG signaling pathways. According to the molecular docking results in Table 3, CASP3, MAPK14, AKT1, CASP8, and MAPK8 is the highest to the lowest order of affinities predicted for the interaction between each of the five protein targets and stemazole.

**Figure 5:**
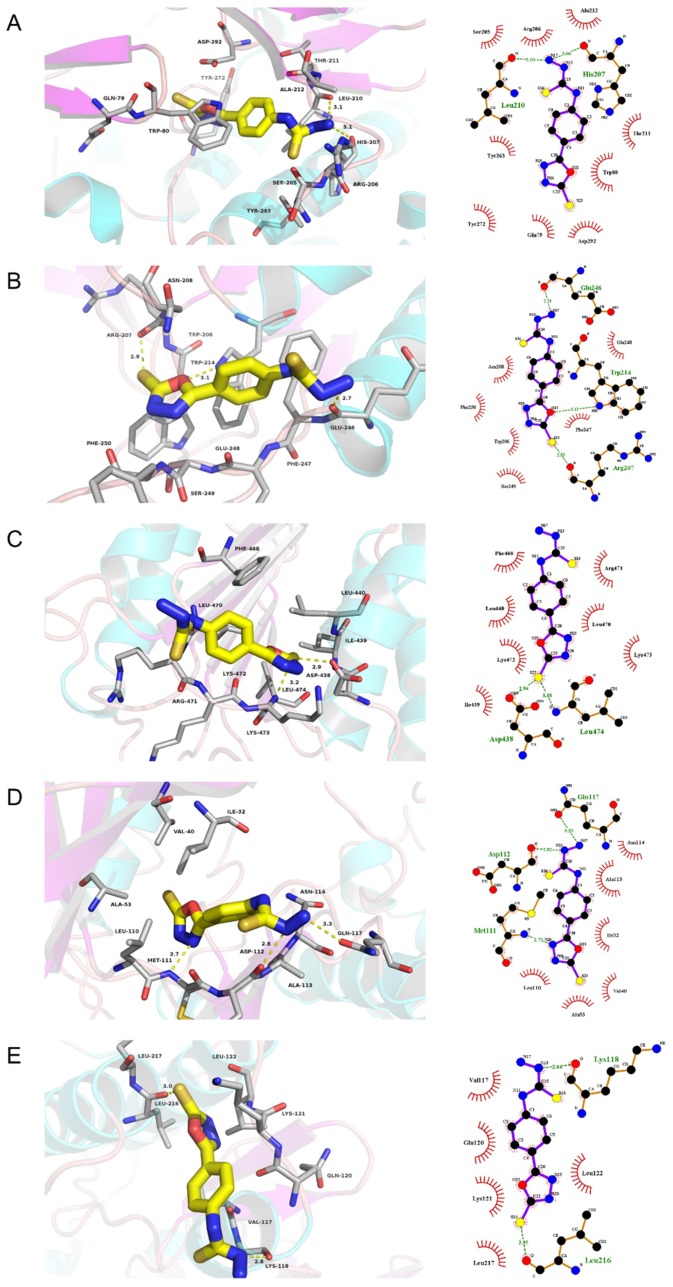
Molecular models of the binding of stemazole to the predicted targets (**A**) AKT1, (**B**) CASP3, (**C**) CASP8, (**D**) MAPK8, and (**E**) MAPK14 shown as 3D diagrams and 2D diagrams.

The docking results in this study demonstrate that the receptor–ligand interaction between ST and AKT1 involves both hydrophobic interactions and polar interactions. As shown in Figure 5A, their interaction is centered on a stable hydrophobic core consisting of several nonpolar residues in AKT1 (Ser205, Arg206, Ala212, Tyr263, Thr211, Trp80, Tyr272, Gln79, and Asp292). In addition, the hydroxyls within the main chains of Leu210 (3.10 Å) and His207 (3.06 Å) form two hydrogen bond contacts with the N17 atom of ST, which further stabilizes the entire interaction region. These interactions enable ST to bind to AKT1.

Moreover, the results in Figure 5B show that ST can bind to CASP3 by forming a hydrophobic interaction with the surrounding residues Asn208, Glu248, Phe250, Trp206, Ser249, and Phe247. ST could form three H-bonds with Glu246 (2.74 Å), Trp214 (3.13 Å), and Arg207 (2.93 Å).

The action modes of ST and CASP8 are shown in Figure 5C. ST binds to a pocket in CASP8, which is comprised of Phe468, Arg471, Leu440, Leu470, Lys472, Lys473, and Ile439. Two hydrogen bonds, ST_S23_:Asp438_OD2_ (2.94 Å) and ST_S23_:Leu474_N_ (3.18 Å), further enhance the interaction between the ligand and the CASP8 protein.

According to the analysis shown in Figure 5D, ST was observed to form hydrophobic interactions with six residues in MAPK8 (Ala113, Asn114, Ile32, Leu110, Ala53, and Val40) and three hydrogen bonds (ST_N17_:Gln117_OE1_ (3.33 Å), ST_N13_:Asp112_O_ (2.82 Å), and ST_N25_:Met111_N_ (2.73 Å)).

As shown in Figure 5E, ST was predicted to interact with MAPK14 via Val117, Gln120, Lys121, Leu217, and Leu122. ST also forms H-bonds with the residues Lys118 (2.84 Å) and Leu216 (2.95 Å).

## 3. Discussion

There is an urgent need to develop new treatment strategies because of the growing crisis resulting from neurodegenerative diseases and the poor efficacy of the existing approved drugs. Stemazole is a novel small molecule with neuroprotective effects, and its therapeutic efficacy in preclinical models of Alzheimer’s disease and Parkinson’s disease has been demonstrated in several studies. Stemazole also protects multiple types of stem cells from apoptosis. Here, a network pharmacology strategy was used to reveal the mechanism underlying the effects of stemazole. Therapeutic targets and the signaling pathways in which they participate were explored by reverse screening, PPI network construction, and pathway enrichment analysis. Information from various online servers and databases was integrated. The results we obtained are very reliable, as they benefitted from multiple information sources and different target identification methods. The specific interactions between stemazole and the targets were verified through molecular docking. Fifty-three signaling pathways and 29 proteins were shown to be involved. Five proteins were identified as the most probable targets. The results could provide a better understanding of the effects of stemazole on the treatment of neurodegenerative diseases and guide further studies of the development of stemazole as a stem cell drug.

We proposed that stemazole plays a therapeutic role in the treatment of neurodegenerative diseases that is mediated by an anti-apoptotic pathway, and the theoretical calculations were carried out on this basis. A similar therapeutic mechanism has been observed for other active small molecules. Pieper and colleagues discovered an aminopropyl carbazole named P7C3, which was utilized in an in vivo screen [25]. A series of P7C3-derived compounds showed neuroprotective activity in multiple models of Alzheimer’s disease [26], Parkinson’s disease [27], and traumatic brain injury [28] by protecting immature neurons from apoptotic cell death and promoting mature neuronal survival. Ye et al. reported a small molecule, 7,8-dihydroxyflavone (DHF), with the ability to protect neurons from apoptosis [29]. DHF has shown promising therapeutic efficacy in rodent models of Alzheimer’s disease [30], Parkinson’s disease [29], Huntington’s disease [31], and amyotrophic lateral sclerosis [32]. Previous experiments have proven that the anti-apoptosis mechanism may serve as the basis of a promising treatment. On the basis of this fact, it is crucial for drug research to explore the properties of drug candidates, especially their underlying molecular mechanisms. The mechanism underlying the effects of stemazole will be discussed in the following section.

The targets of stemazole were enriched in the MAPK signaling pathway, which was indicated by the KEGG pathway enrichment analysis. Previous studies have shown that the activation of the MAPK signaling pathway is highly correlated with the occurrence and development of neurodegenerative diseases. In the pathogenesis of AD, the MAPK pathway contributes to disease progression by inducing neuronal apoptosis, the transcription and activation of β- and γ-secretases, and the phosphorylation of Amyloid-beta precursor protein (APP) and tau. The role of the MAPK signaling pathway in PD involves the induction of neuronal death and neuroinflammatory responses associated with the levels of α-synuclein and functional deficiencies in parkin and Protein/nucleic acid deglycase DJ-1 [33]. The MAPK signaling pathway leads to apoptosis in both AD and PD, which suggests the importance of the anti-apoptosis ability of stemazole.

Two methods, centrality analysis of the PPI network and KEGG pathway analysis, were used individually to identify the core targets. According to the centrality and the node degree of the protein–protein interaction network, we identified the essential proteins, including MAPK8, AKT1, MAPK14, FYN, CASP3, ESR1, AR, and CASP8. Nodes with high centralities and node degrees often play the most important roles in the network. Among the top 20 enriched KEGG pathways, MAPK8, AKT1, MAPK14, CASP3, CASP8, TNF, and CASP9 were considered important because of their high frequency of involvement. The results of the two methods were not exactly the same. Proteins identified as central targets by both methods were selected for further analysis at the molecular level.

Five targets, MAPK8, AKT1, MAPK14, CASP3, and CASP8, were selected for the molecular docking studies. Stemazole showed good affinity towards the five targets, which validated the network pharmacology results. These targets play important roles in the pathophysiology of neurodegenerative diseases. Akt1 is highly expressed in the nervous system and is critical for the survival of neuronal cells [34]. Neuroprotective effects were observed after AKT1 kinase activity was enhanced, and pAKT1 was upregulated in cellular and animal models of neurodegenerative diseases [35]. JNK1 (MAPK8) is the main c-Jun N-terminal kinase involved in brain physiological activity [36]. Activated JNK plays an important role in amyloid plaque formation, Aβ deposition, the phosphorylation of tau, neuroinflammation, and Aβ-induced synaptic dysfunction [37], all of which are physiological processes involved in AD [38]. p38-α (MAPK14) mediates oxidative stress and leads to the aggregation of the tau protein [39]. After inhibiting p38 in rodent models of AD, the apoptosis of nerve cells and damage to cognitive function were greatly reduced [40]. In addition, p38 can also lead to the degeneration and death of dopaminergic neurons in Parkinson’s disease [41]. Caspase-8 and caspase-3 are two cysteine aspartate-specific proteases. Apoptotic neurons act as part of initiator and executioner cascades in apoptotic cascades [42]. The experimental results indicated that caspase-8 can mediate neuronal death induced by beta-amyloid protein [43]. As a downstream effector of caspase-8, caspase-3 is the core executioner caspase. Activation and increased expression of caspase-3 were observed in the brain in AD. Caspase-3 is also involved in APP proteolysis and Aβ peptide formation [44]. Through genetic intervention via caspase-3, dopaminergic neurons can be protected against cell death induced by oxidative stress, which suggests that caspase-3 is an important target for the prevention of the progression of PD [45].

The results of the network analysis provide a theoretical basis and important information that could be useful for elucidating the mechanisms underlying the therapeutic effects of stemazole and identifying potential targets, but the experimental verification of the targets and specific interactions will be necessary in the future.

## 4. Materials and Methods

**Figure 6:**
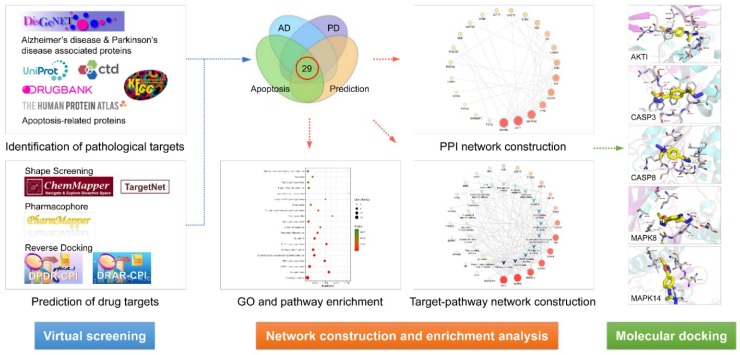
The experimental flow of this study. AD, Alzheimer’s disease; PD, Parkinson’s disease; PPI, protein–protein interaction; GO, gene ontology.

The experimental flow is shown in Figure 6.

### 4.1. Identification of Pathological Targets

Targets associated with AD and PD were identified using the DisGeNET database v6.0 (http://www.disgenet.org/) [46]. Apoptosis-related proteins and genes were identified using the Human Protein Atlas (https://www.proteinatlas.org/) [47], the DrugBank database (https://www.drugbank.ca/) [48], UniProt (https://www.uniprot.org/) [49], the Comparative Toxicogenomics Database (CTD, http://ctdbase.org/) [50], and the Kyoto Encyclopedia of Genes and Genomes (KEGG, https://www.kegg.jp/) [51].

### 4.2. Virtual Screening of Drug Targets

The three-dimensional structure of stemazole was built and optimized in Gaussian 09W software [52] at the DFT-B3LYP/6-311G (d, p) level. The optimized structure was saved in mol2 and sdf format prior to the screening processes. The SMILES string of stemazole was generated using Advanced Chemistry Development/ChemSketch software [53].

According their theoretical basis, reverse screening methods are classified into one of three types: shape screening, pharmacophore screening, or reverse docking [54]. Each method has its own characteristics in terms of algorithms and programs. Each database contains only a small number of protein targets. Therefore, several servers were used to combine prediction methods to utilize a wide range of targets and obtain reliable results.

The 3D structural file or SMILES string was submitted to the PharmMapper (http://lilab-ecust.cn/pharmmapper/) [55], DRAR-CPI (http://cpi.bio-x.cn/drar/) [56], DPDR-CPI (http://cpi.bio-x.cn/dpdr/) [57], TargetNet (http://targetnet.scbdd.com/) [58], and ChemMapper (http://lilab-ecust.cn/chemmapper/) [59] servers to predict the targets of stemazole. *Homo sapiens* was chosen as the organism.

After adding the targets together and deleting the duplicates, the obtained predicted targets were assumed to be pathological targets. Then, the therapeutic targets of stemazole relevant to its effects on AD and PD via its anti-apoptosis mechanism were preliminarily determined.

### 4.3. PPI Network Construction and Analysis

The identified targets were uploaded to the STRING database v11.0 (http://string-db.org) [60] to obtain the protein–protein interaction information, including the physical and functional associations. Protein interactions with a confidence score of 0.9 or higher were exported in TSV format. The PPI network was visualized by Cytoscape v3.7.1 [61]. Network Analyzer [62] was utilized for calculating the network topology parameters, in which the network was treated as undirected. The CytoNCA plugin (v2.1.6) [63] was used to measure the topology scores of the nodes, including the betweenness, closeness, and subgraph centrality. The option “without weight” was selected.

### 4.4. GO and KEGG Pathway Enrichment Analyses

The gene ontology and KEGG pathway enrichment analyses were carried out using the web server g: Profiler (https://biit.cs.ut.ee/gprofiler) [64] and visualized by the OmicShare cloud platform as a bubble chart (http://www.omicshare.com/). The target-pathway network was constructed in Cytoscape v3.7.1.

### 4.5. Molecular Docking

The X-ray crystal structures of the targets (http://www.rcsb.org/) [65,66], including AKT1 (PDB ID: 6HHF), CASP3 (PDB ID: 5IAG), CASP8 (PDB ID: 3KJN), MAPK8 (PDB ID: 3O2M), and MAPK14 (PDB ID: 5ETC), were obtained from the Protein Data Bank (PDB). The water molecules and hetero atoms were then removed from the proteins using Chimera 1.13.1 [67]. The protein receptor files and ligand file were processed using AutoDock Tools 1.5.6, including adding hydrogen atoms, calculating and adding Gasteiger charges, merging nonpolar hydrogen atoms, and setting rotatable torsion bonds. Then, they were converted to pdbqt format. The parameters of the grid box are shown in Table 4. Each grid box was centered on and encompassed the entirety of the active site. Molecular docking between stemazole and the core targets was carried out using AutoDock 4.2 software [68] with the Lamarckian genetic algorithm. The number of GA runs was set to 50, and other parameters were set to default values. The binding mode with the lowest binding energy in the cluster with the most conformations was selected for further analysis. The interactions between stemazole and the predicted targets were visualized and displayed as 3D diagrams and 2D diagrams by using PyMOL 1.8 and ligplus.

## 5. Conclusions

The potential targets and molecular mechanisms underlying the effects of stemazole against AD and PD mediated by anti-apoptosis were systematically investigated by network pharmacology. The key pathway involved in the neuroprotective effects of stemazole is the MAPK signaling pathway, and five key targets were identified, including MAPK8, AKT1, MAPK14, CASP3, and CASP8. In summary, the presented findings may inspire and guide further pharmacological studies of the effects of stemazole against neurodegenerative diseases.

## Figures and Tables

**Table 1:** Putative therapeutic targets of stemazole mediating its effects against neurodegenerative diseases.

No.	Symbol	Protein Name	Database
1	ABL1	Tyrosine-protein kinase ABL1	DRAR-CPI, DPDR-CPI, PharmMapper
2	ACHE	Acetylcholinesterase	ChemMapper
3	AKT1	RAC-alpha serine/threonine-protein kinase	ChemMapper
4	AR	Androgen receptor	DPDR-CPI, ChemMapper
5	CASP3	Caspase-3	PharmMapper
6	CASP8	Caspase-8	DPDR-CPI
7	CASP9	Caspase-9	TargetNet
8	CDK5	Cyclin-dependent-like kinase 5	ChemMapper
9	CSF1R	Macrophage colony-stimulating factor 1 receptor	DPDR-CPI
10	DAPK1	Death-associated protein kinase 1	PharmMapper
11	DRD3	D(3) dopamine receptor	ChemMapper
12	ESR1	Estrogen receptor	DRAR-CPI, PharmMapper, ChemMapper, TargetNet
13	FGF2	Fibroblast growth factor 2	ChemMapper
14	FYN	Tyrosine-protein kinase Fyn	DPDR-CPI
15	GAPDH	Glyceraldehyde-3-phosphate dehydrogenase	ChemMapper
16	HGF	Hepatocyte growth factor	ChemMapper
17	HMGCR	3-hydroxy-3-methylglutaryl-coenzyme A reductase	PharmMapper
18	IGF1R	Insulin-like growth factor 1 receptor	DRAR-CPI
19	INSR	Insulin receptor	PharmMapper
20	MAPK14	Mitogen-activated protein kinase 14	PharmMapper, ChemMapper
21	MAPK8	Mitogen-activated protein kinase 8	DPDR-CPI, PharmMapper, ChemMapper
22	MAPK8IP1	C-Jun-amino-terminal kinase-interacting protein 1	ChemMapper
23	MMP9	Matrix metalloproteinase-9	DPDR-CPI
24	PIK3CG	Phosphatidylinositol 4,5-bisphosphate 3-kinase catalytic subunit gamma isoform	DPDR-CPI, TargetNet
25	PPARD	Peroxisome proliferator-activated receptor delta	DRAR-CPI
26	PPARG	Peroxisome proliferator-activated receptor gamma	DRAR-CPI, ChemMapper
27	PPIF	Peptidyl-prolyl cis-trans isomerase F, mitochondrial	ChemMapper
28	PTGS2	Prostaglandin G/H synthase 2	ChemMapper, TargetNet
29	TNF	Tumor necrosis factor	DRAR-CPI

**Table 2:** Topological parameters of the targets.

	Degree	Subgragh	Betweenness	Closeness
MAPK8	9	35.681553	164.86667	0.6111111
AKT1	9	33.13798	71.3	0.5945946
MAPK14	8	40.777042	100.933334	0.52380955
FYN	6	23.114271	116.72222	0.5
CASP3	6	20.888252	63.6	0.5
ESR1	5	16.392994	46.566666	0.5
AR	4	15.240643	53.433334	0.47826087
CASP8	4	9.148983	11.6	0.4680851
ABL1	4	14.081305	18	0.41509435
TNF	6	13.089347	8.866667	0.4489796
CASP9	3	7.837092	3.4444444	0.43137255
CDK5	3	5.526705	8.5	0.37931034
INSR	2	3.5084944	42	0.4
IGF1R	2	2.9850514	0	0.4
PPARG	2	2.9773748	0	0.3859649
HGF	2	3.2480063	8	0.36666667
GAPDH	2	4.990251	5.8333335	0.36065573
PIK3CG	2	5.3232713	2.3333333	0.33846155
PTGS2	1	1.9571668	0	0.3859649
DAPK1	1	2.1390505	0	0.33846155
MMP9	1	1.957167	0	0.33846155
FGF2	1	1.6149884	0	0.33846155
MAPK8IP1	1	2.5664337	0	0.28947368

**Table 3:** Results of molecular docking between stemazole (ST) and the predicted target.

Receptors	Binding Energy (ΔG)/kcal·moL^−1^	Inhibit Constant (Ki)/μM
CASP3	−7.45	3.45
MAPK14	−7.21	5.17
AKT1	−7.04	6.86
CASP8	−6.48	17.92
MAPK8	−6.38	20.9

**Table 4:** Parameters of the grid box in molecular docking.

Targets	PDB ID	Grid Center			Npts	Spacing
AKT1	6HHF	5.230	2.277	20.620	60 60 60	0.375
CASP3	5IAG	8.173	−18.986	−21.032	60 60 60	0.375
CASP8	3KJN	−5.713	17.721	16.597	60 60 60	0.375
MAPK8	3O2M	17.929	107.605	53.797	60 60 60	0.375
MAPK14	5ETC	5.881	76.862	22.002	60 60 60	0.375

## Supplementary Materials

Supplementary materials can be found at https://www.mdpi.com/1422-0067/21/2/427/s1. Table S1: Targets associated with Alzheimer’s disease and Parkinson’s disease, Table S2: Predicted targets of stemazole, Table S3: The results of the GO and KEGG pathway enrichment.

## Abbreviations

GO	gene ontology
KEGG	Kyoto Encyclopedia of Genes and Genomes
AKT1	RAC-alpha serine/threonine-protein kinase
CASP3	caspase-3
CASP8	caspase-8
MAPK8	mitogen-activated protein kinase 8
MAPK14	mitogen-activated protein kinase 14
AD	Alzheimer’s disease
PD	Parkinson’s disease
ST	Stemazole
Aβ	beta-amyloid
MPTP	1-methyl-4-phenyl-1,2,3,6-tetrahydropyridine
ABL1	Tyrosine-protein kinase ABL1
ACHE	Acetylcholinesterase
AR	Androgen receptor
CASP9	Caspase-9
CDK5	Cyclin-dependent-like kinase 5
CSF1R	Macrophage colony-stimulating factor 1 receptor
DAPK1	Death-associated protein kinase 1
DRD3	D(3) dopamine receptor
ESR1	Estrogen receptor
FGF2	Fibroblast growth factor 2
FYN	Tyrosine-protein kinase Fyn
GAPDH	Glyceraldehyde-3-phosphate dehydrogenase
HGF	Hepatocyte growth factor
HMGCR	3-hydroxy-3-methylglutaryl-coenzyme A reductase
IGF1R	Insulin-like growth factor 1 receptor
INSR	Insulin receptor
MAPK8IP1	C-Jun-amino-terminal kinase-interacting protein 1
MMP9	Matrix metalloproteinase-9
PIK3CG	Phosphatidylinositol 4,5-bisphosphate 3-kinase catalytic subunit gamma isoform
PPARD	Peroxisome proliferator-activated receptor delta
PPARG	Peroxisome proliferator-activated receptor gamma
PPIF	Peptidyl-prolyl cis-trans isomerase F, mitochondrial
PTGS2	Prostaglandin G/H synthase 2
TNF	Tumor necrosis factor
PPI	Protein–protein interaction
DHF	7,8-dihydroxyflavone

## References

[1] Friedlander R.M. (2003). Mechanisms of disease: Apoptosis and caspases in neurodegenerative diseases. N. Engl. J. Med..

[2] Wyss-Coray T. (2016). Ageing, neurodegeneration and brain rejuvenation. Nature.

[3] Reitz C., Brayne C., Mayeux R. (2011). Epidemiology of Alzheimer disease. Nat. Rev. Neurol..

[4] Kalia L.V., Lang A.E. (2015). Parkinson’s disease. Lancet.

[5] Patterson C. (2018). The State of the Art of Dementia Research: New Frontiers.

[6] Dorsey E.R., Bloem B.R. (2018). The Parkinson Pandemic-A Call to Action. JAMA Neurol..

[7] Bachurin S.O., Bovina E.V., Ustyugov A.A. (2017). Drugs in Clinical Trials for Alzheimer’s Disease: The Major Trends. Med. Res. Rev..

[8] Fernandez H.H. (2015). 2015 Update on Parkinson disease. Clevel. Clin. J. Med..

[9] Cummings J., Aisen P.S., DuBois B., Frölich L., Jack C.R., Jones R.W., Morris J.C., Raskin J., Dowsett S.A., Scheltens P. (2016). Drug development in Alzheimer’s disease: The path to 2025. Alzheimer’s Res. Ther..

[10] Charvin D., Medori R., Hauser R.A., Rascol O. (2018). Therapeutic strategies for Parkinson disease: Beyond dopaminergic drugs. Nat. Rev. Drug Discov..

[11] Palop J.J., Chin J., Mucke L. (2006). A network dysfunction perspective on neurodegenerative diseases. Nature.

[12] Lev N., Melamed E., Offen D. (2003). Apoptosis and Parkinson’s disease. Prog. Neuro Psychopharmacol. Biol. Psychiatry.

[13] Singh S., Srivastava A., Srivastava P., Dhuriya Y.K., Pandey A., Kumar D., Rajpurohit C.S. (2016). Advances in Stem Cell Research—A Ray of Hope in Better Diagnosis and Prognosis in Neurodegenerative Diseases. Front. Mol. Biosci..

[14] Stoddard-Bennett T., Pera R.R. (2019). Treatment of Parkinson’s Disease through Personalized Medicine and Induced Pluripotent Stem Cells. Cells.

[15] Yue C., Jing N. (2015). The promise of stem cells in the therapy of Alzheimer’s disease. Transl. Neurodegener..

[16] Han F., Baremberg D., Gao J., Duan J., Lu X., Zhang N., Chen Q. (2015). Development of stem cell-based therapy for Parkinson’s disease. Transl. Neurodegener..

[17] Sun Y., Wang W., Sun Y., Han M. (2011). Synthesis and biological evaluation of a novel human stem/progenitor cells proliferation activator: 4-(4-(5-mercapto-1,3,4-oxadiazol-2-yl)phenyl) thiosemicarbazide (Stemazole). Eur. J. Med. Chem..

[18] Sun Y., Zhang X., Li H., Xu S., Zhang X., Liu Y., Han M., Wen J. (2018). Stemazole promotes survival and preserves stemness in human embryonic stem cells. FEBS J..

[19] Han M., Liu Y., Tan Q., Zhang B., Wang W., Liu J., Zhang X.-J., Wang Y.-Y., Zhang J.-M. (2011). Therapeutic efficacy of stemazole in a beta-amyloid injection rat model of Alzheimer’s disease. Eur. J. Pharmacol..

[20] Guo Z., Xu S., Du N., Liu J., Huang Y., Han M. (2016). Neuroprotective effects of stemazole in the MPTP-induced acute model of Parkinson’s disease: Involvement of the dopamine system. Neurosci. Lett..

[21] Li H., Tan Q., Zhang Y., Zhang J., Zhao C., Lu S., Qiao J., Han M. (2019). Pharmacokinetics and absolute oral bioavailability of stemazole by UPLC-MS/MS and its bio-distribution through tritium labeling. Drug Test. Anal..

[22] Hopkins A.L. (2008). Network pharmacology: The next paradigm in drug discovery. Nat. Chem. Biol..

[23] Park M., Park S.-Y., Lee H.-J., Kim C.-E. (2018). A Systems-Level Analysis of Mechanisms of Platycodon grandiflorum Based on A Network Pharmacological Approach. Molecules.

[24] Boezio B., Audouze K., Ducrot P., Taboureau O. (2017). Network-based Approaches in Pharmacology. Mol. Inform..

[25] Pieper A.A., Xie S., Capota E., Estill S.J., Zhong J., Long J.M., Becker G.L., Huntington P., Goldman S.E., Shen C.-H. (2010). Discovery of a Proneurogenic, Neuroprotective Chemical. Cell.

[26] Voorhees J.R., Remy M.T., Cintron-Perez C.J., El Rassi E., Khan M.Z., Dutca L.M., Yin T.C., McDaniel L.N., Williams N.S., Brat D.J. (2018). (-)-P7C3-S243 Protects a Rat Model of Alzheimer’s Disease From Neuropsychiatric Deficits and Neurodegeneration Without Altering Amyloid Deposition or Reactive Glia. Biol. Psychiatry.

[27] De Jesus-Cortes H., Xu P., Drawbridge J., Estill S.J., Huntington P., Tran S., Britt J., Tesla R., Morlock L., Naidoo J. (2012). Neuroprotective efficacy of aminopropyl carbazoles in a mouse model of Parkinson disease. Proc. Natl. Acad. Sci. USA.

[28] Yin T.C., Britt J.K., De Jesus-Cortes H., Lu Y., Genova R.M., Khan M.Z., Voorhees J.R., Shao J., Katzman A.C., Huntington P.J. (2014). P7C3 Neuroprotective Chemicals Block Axonal Degeneration and Preserve Function after Traumatic Brain Injury. Cell Rep..

[29] Jang S.-W., Liu X., Yepes M., Shepherd K.R., Miller G.W., Liu Y., Wilson W.D., Xiao G., Blanchi B., Sun Y.E. (2010). A selective TrkB agonist with potent neurotrophic activities by 7,8-dihydroxyflavone. Proc. Natl. Acad. Sci. USA.

[30] Zhang Z., Liu X., Schroeder J.P., Chan C.-B., Song M., Yu S.P., Weinshenker D., Ye K. (2014). 7,8-Dihydroxyflavone Prevents Synaptic Loss and Memory Deficits in a Mouse Model of Alzheimer’s Disease. Neuropsychopharmacology.

[31] Jiang M., Peng Q., Liu X., Jin J., Hou Z., Zhang J., Mori S., Ross C.A., Ye K., Duan W. (2013). Small-molecule TrkB receptor agonists improve motor function and extend survival in a mouse model of Huntingtons disease. Hum. Mol. Genet..

[32] Korkmaz O.T., Aytan N., Carreras I., Choi J.-K., Kowall N.W., Jenkins B.G., Dedeoglu A. (2014). 7,8-Dihydroxyflavone improves motor performance and enhances lower motor neuronal survival in a mouse model of amyotrophic lateral sclerosis. Neurosci. Lett..

[33] Kim E.K., Choi E.-J. (2010). Pathological roles of MAPK signaling pathways in human diseases. Biochim. Biophys. Acta Mol. Basis Dis..

[34] Griffin R.J., Moloney A., Kelliher M., Johnston J.A., Ravid R., Dockery P., O’Connor R., O’Neill C. (2005). Activation of Akt/PKB, increased phosphorylation of Akt substrates and loss and altered distribution of Akt and PTEN are features of Alzheimer’s disease pathology. J. Neurochem..

[35] Ahmad F., Nidadavolu P., Durgadoss L., Ravindranath V. (2014). Critical cysteines in Akt1 regulate its activity and proteasomal degradation: Implications for neurodegenerative diseases. Free Radic. Biol. Med..

[36] Coffey E.T., Smiciene G., Hongisto V., Cao J., Brecht S., Herdegen T., Courtney M.J. (2002). c-Jun N-terminal protein kinase (JNK) 2/3 is specifically activated by stress, mediating c-Jun activation, in the presence of constitutive JNK1 activity in cerebellar neurons. J. Neurosci..

[37] Zhou Q., Wang M., Du Y., Zhang W., Bai M., Zhang Z., Li Z., Miao J. (2015). Inhibition of c-Jun N-Terminal Kinase Activation Reverses Alzheimer Disease Phenotypes in APPswe/PS1dE9 Mice. Ann. Neurol..

[38] Mehan S., Meena H., Sharma D., Sankhla R. (2011). JNK: A Stress-Activated Protein Kinase Therapeutic Strategies and Involvement in Alzheimer’s and Various Neurodegenerative Abnormalities. J. Mol. Neurosci..

[39] Giraldo E., Lloret A., Fuchsberger T., Viña J. (2014). Aβ and tau toxicities in Alzheimer’s are linked via oxidative stress-induced p38 activation: Protective role of vitamin E. Redox Biol..

[40] Guo J., Cheng J., North B.J., Wei W. (2017). Functional analyses of major cancer-related signaling pathways in Alzheimer’s disease etiology. Biochim. Biophys. Acta. Rev. Cancer.

[41] Corti O., Hampe C., Koutnikova H., Darios F., Jacquier S., Prigent A., Robinson J.C., Pradier L., Ruberg M., Mirande M. (2003). The p38 subunit of the aminoacyl-tRNA synthetase complex is a Parkin substrate: Linking protein biosynthesis and neurodegeneration. Hum. Mol. Genet..

[42] Louneva N., Cohen J.W., Han L.Y., Talbot K., Wilson R.S., Bennett D.A., Trojanowski J.Q., Arnold S.E. (2008). Caspase-3 is enriched in postsynaptic densities and increased in Alzheimer’s disease. Am. J. Pathol..

[43] Ivins K.J., Thornton P.L., Rohn T.T., Cotman C.W. (1999). Neuronal apoptosis induced by beta-amyloid is mediated by caspase-8. Neurobiol. Dis..

[44] D’Amelio M., Cavallucci V., Middei S., Marchetti C., Pacioni S., Ferri A., Diamantini A., De Zio D., Carrara P., Battistini L. (2011). Caspase-3 triggers early synaptic dysfunction in a mouse model of Alzheimer’s disease. Nat. Neurosci..

[45] Kim J.S., Ha J.Y., Yang S.J., Son J.H. (2018). A Novel Non-Apoptotic Role of Procaspase-3 in the Regulation of Mitochondrial Biogenesis Activators. J. Cell. Biochem..

[46] Pinero J., Bravo A., Queralt-Rosinach N., Gutierrez-Sacristan A., Deu-Pons J., Centeno E., Garcia-Garcia J., Sanz F., Furlong L.I. (2017). DisGeNET: A comprehensive platform integrating information on human disease-associated genes and variants. Nucleic Acids Res..

[47] Uhlen M., Oksvold P., Fagerberg L., Lundberg E., Jonasson K., Forsberg M., Zwahlen M., Kampf C., Wester K., Hober S. (2010). Towards a knowledge-based Human Protein Atlas. Nat. Biotechnol..

[48] Wishart D.S., Feunang Y.D., Guo A.C., Lo E.J., Marcu A., Grant J.R., Sajed T., Johnson D., Li C., Sayeeda Z. (2018). DrugBank 5.0: A major update to the DrugBank database for 2018. Nucleic Acids Res..

[49] Bateman A., Martin M.-J., Orchard S., Magrane M., Alpi E., Bely B., Bingley M., Britto R., Bursteinas B., Busiello G. (2019). UniProt: A worldwide hub of protein knowledge. Nucleic Acids Res..

[50] Davis A.P., Grondin C.J., Johnson R.J., Sciaky D., McMorran R., Wiegers J., Wiegers T.C., Mattingly C.J. (2019). The Comparative Toxicogenomics Database: Update 2019. Nucleic Acids Res..

[51] Kanehisa M., Goto S. (2000). KEGG: Kyoto Encyclopedia of Genes and Genomes. Nucleic Acids Res..

[52] Frisch M.J., Trucks G.W., Schlegel H.B., Scuseria G.E., Robb M.A., Cheeseman J.R., Scalmani G., Barone V., Petersson G.A., Nakatsuji H. (2009). Gaussian 09 Revision A.1.

[53] ACD/Labs (2019). ACD/ChemSketch, Version 2018.2.

[54] Huang H., Zhang G., Zhou Y., Lin C., Chen S., Lin Y., Mai S., Huang Z. (2018). Reverse Screening Methods to Search for the Protein Targets of Chemopreventive Compounds. Front. Chem..

[55] Wang X., Shen Y., Wang S., Li S., Zhang W., Liu X., Lai L., Pei J., Li H. (2017). PharmMapper 2017 update: A web server for potential drug target identification with a comprehensive target pharmacophore database. Nucleic Acids Res..

[56] Luo H., Chen J., Shi L., Mikailov M., Zhu H., Wang K., He L., Yang L. (2011). DRAR-CPI: A server for identifying drug repositioning potential and adverse drug reactions via the chemical-protein interactome. Nucleic Acids Res..

[57] Luo H., Zhang P., Cao X.H., Du D., Ye H., Huang H., Li C., Qin S., Wan C., Shi L. (2016). DPDR-CPI, a server that predicts Drug Positioning and Drug Repositioning via Chemical-Protein Interactome. Sci. Rep..

[58] Yao Z.-J., Dong J., Che Y.-J., Zhu M.-F., Wen M., Wang N.-N., Wang S., Lu A.-P., Cao D.-S. (2016). TargetNet: A web service for predicting potential drug-target interaction profiling via multi-target SAR models. J. Comput. Aided Mol. Des..

[59] Gong J., Cai C., Liu X., Ku X., Jiang H., Gao D., Li H. (2013). ChemMapper: A versatile web server for exploring pharmacology and chemical structure association based on molecular 3D similarity method. Bioinformatics.

[60] Szklarczyk D., Franceschini A., Wyder S., Forslund K., Heller D., Huerta-Cepas J., Simonovic M., Roth A., Santos A., Tsafou K.P. (2015). STRING v10: Protein-protein interaction networks, integrated over the tree of life. Nucleic Acids Res..

[61] Shannon P., Markiel A., Ozier O., Baliga N.S., Wang J.T., Ramage D., Amin N., Schwikowski B., Ideker T. (2003). Cytoscape: A software environment for integrated models of biomolecular interaction networks. Genome Res..

[62] Assenov Y., Ramirez F., Schelhorn S.-E., Lengauer T., Albrecht M. (2008). Computing topological parameters of biological networks. Bioinformatics.

[63] Tang Y., Li M., Wang J., Pan Y., Wu F.-X. (2015). CytoNCA: A cytoscape plugin for centrality analysis and evaluation of protein interaction networks. Biosystems.

[64] Raudvere U., Kolberg L., Kuzmin I., Arak T., Adler P., Peterson H., Vilo J. (2019). g:Profiler: A web server for functional enrichment analysis and conversions of gene lists (2019 update). Nucleic Acids Res..

[65] Berman H.M., Westbrook J., Feng Z., Gilliland G., Bhat T.N., Weissig H., Shindyalov I.N., Bourne P.E. (2000). The Protein Data Bank. Nucleic Acids Res..

[66] Burley S.K., Berman H.M., Bhikadiya C., Bi C., Chen L., Di Costanzo L., Christie C., Dalenberg K., Duarte J.M., Dutta S. (2018). RCSB Protein Data Bank: Biological macromolecular structures enabling research and education in fundamental biology, biomedicine, biotechnology and energy. Nucleic Acids Res..

[67] Pettersen E.F., Goddard T.D., Huang C.C., Couch G.S., Greenblatt D.M., Meng E.C., Ferrin T.E. (2004). UCSF chimera—A visualization system for exploratory research and analysis. J. Comput. Chem..

[68] Morris G.M., Huey R., Lindstrom W., Sanner M.F., Belew R.K., Goodsell D.S., Olson A.J. (2009). AutoDock4 and AutoDockTools4: Automated Docking with Selective Receptor Flexibility. J. Comput. Chem..

